# Alpha test results for a Housing First eLearning strategy: the value of multiple qualitative methods for intervention design

**DOI:** 10.1186/s40814-017-0187-y

**Published:** 2017-10-31

**Authors:** Emily Q. Ahonen, Dennis P. Watson, Erin L. Adams, Alan McGuire

**Affiliations:** 1 Richard M. Fairbanks School of Public Health, Indiana University-Purdue University Indianapolis, 714 N. Senate Ave, Indianapolis, IN 46202 USA; 2 Department of Psychology, Indiana University Purdue University-Indianapolis, 420 N Blackford St., Indianapolis, IN 46202 USA; 3 Richard L. Roudebush VA, 1481 W. 10th St., Indianapolis, IN 46202 USA

**Keywords:** Implementation strategy, Intervention development, Housing First, eLearning, Digital badging, Community of practice, Narrative storytelling, Alpha test

## Abstract

**Background:**

Detailed descriptions of implementation strategies are lacking, and there is a corresponding dearth of information regarding methods employed in implementation strategy development. This paper describes methods and findings related to the alpha testing of eLearning modules developed as part of the Housing First Technical Assistance and Training (HFTAT) program’s development. Alpha testing is an approach for improving the quality of a product prior to beta (i.e., real world) testing with potential applications for intervention development.

**Methods:**

Ten participants in two cities tested the modules. We collected data through (1) a structured log where participants were asked to record their experiences as they worked through the modules; (2) a brief online questionnaire delivered at the end of each module; and (3) focus groups.

**Results:**

The alpha test provided useful data related to the acceptability and feasibility of eLearning as an implementation strategy, as well as identifying a number of technical issues and bugs. Each of the qualitative methods used provided unique and valuable information. In particular, logs were the most useful for identifying technical issues, and focus groups provided high quality data regarding how the intervention could best be used as an implementation strategy.

**Conclusions:**

Alpha testing was a valuable step in intervention development, providing us an understanding of issues that would have been more difficult to address at a later stage of the study. As a result, we were able to improve the modules prior to pilot testing of the entire HFTAT. Researchers wishing to alpha test interventions prior to piloting should balance the unique benefits of different data collection approaches with the need to minimize burdens for themselves and participants.

**Electronic supplementary material:**

The online version of this article (10.1186/s40814-017-0187-y) contains supplementary material, which is available to authorized users.

## Background

Closing the healthcare research-practice gap requires sound strategies for implementing evidence-based practices; however, just like with intervention development [1, 2], there is a paucity of information regarding empirical methods for implementation strategy development. This paucity may contribute to the lack of detailed implementation strategy descriptions [3, 4], particularly when it comes to complex strategies comprising two or more discrete ones [5–7]. We address the gap in implementation strategy development and explication by describing the first phase of a study aimed at developing the Housing First Technical Assistance and Training (HFTAT) program, a 6-month strategy developed to overcome noted barriers to implementation of the Housing First (HF) permanent supportive housing intervention.

HF is an evidence-based practice designed to serve individuals experiencing chronic homelessness and who also have dually diagnosed mental health and substance use disorders [8, 9]. Our community partner, the Midwest Harm Reduction Institute (MHRI), has delivered face-to-face training and technical assistance to support programs implementing HF for more than a decade. Together, we developed the idea for the HFTAT as a distance-based implementation strategy to increase the MHRI’s capacity to deliver services to more programs over a greater distance [10]. The primary components of the HFTAT include (1) four eLearning (online training) modules^1^ (Introduction to the HF Philosophy, Housing Case Management, Strategies for Engaging Consumers, and Running a Housing First Program) for administrator and staff training; (2) an online community of practice (CoP; i.e., a webpage where HF professionals can interact with the goal of improving their individual practice; the CoP developed for this study can be found at http://housingfirstpracticecommunity.weebly.com); (3) distance-based technical assistance with MHRI staff (including weekly 1-h phone meetings and fidelity monitoring and feedback); and (4) an implementation manual/guide. Training completion is asynchronous and self-paced (within specified parameters), and staff are assigned specific modules based on position (i.e., administrators take all four modules, clinical and case management staff take the first three modules, and all other staff take the first module only). A detailed description of the HFTAT and the design of the larger study is included in a previously published protocol article [11].

**Fig. 1:**
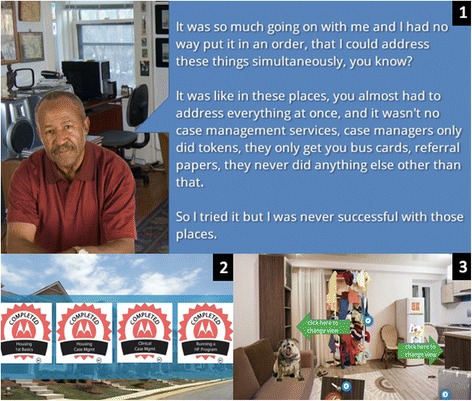
Screen shots from eLearning modules. (Image 1) Selection from a case-based client narrative. (Image 2) Digital badges provided at the end of each module. (Image 3) Interactive home visit activity that asks user to explore a client’s apartment and identify issues needing attention

We designed the HFTAT to overcome a number of HF implementation barriers, many of which are rooted in the intervention’s reliance on complex skills and high levels of coordination among a variety of individuals, organizations, and systems [11]. In addition to providing basic HF knowledge and skills, we designed the eLearning modules to overcome one of the most significant of these barriers, *staff resistance to harm reduction* [12–14]. Harm reduction is a critical component of HF that works with substance users on their own terms [15], which has met resistance in the substance abuse treatment field due to the pervasiveness of abstinence-only attitudes [16, 17]. To address this resistance, we weaved *case-based narratives* (e.g., real stories of people living and working in HF programs) throughout the modules due to narrative storytelling’s potential to overcome attitudinal barriers to the learning and integration of new information [18–21]. Other key components of the modules that we integrated to facilitate meaningful and engaging learning include *cognitively effective design* (e.g., breaking topics into manageable, learner controlled chunks delivered through a mix of audio, images, text, and video) [22]; *opportunities to apply knowledge gained* through challenges, activities, and assessments with feedback; *opportunities for reflection* on prior work through activities and connections to the CoP in order to challenge assumptions and support conceptual change [22]; and *provision of digital badges* (an alternative online credentialing mechanism with theoretical potential to motivate learners [23]^2^) provided as an incentive at the completion of each module. Figure 1 includes screenshots from the training demonstrating several of these approaches.

The purpose of this study phase was to conduct an alpha test of the modules before piloting the entire HFTAT. An alpha test is essentially an approach for improving the quality of a product prior to beta (i.e., practical application) testing. A primary difference between these two stages is that alpha testing is usually carried out among a small convenience sample, while beta testing is conducted within a representative sample of actual customers/users [24, 25]. The purpose of the alpha test was to (1) determine the acceptability of the eLearning modules and feasibility of eLearning as a HF training modality and to (2) identify any technical issues (i.e., “bugs”) prior to pilot (i.e., beta) testing the entire strategy among a sample of programs seeking to implement the HF model. As a contribution to the nascent literature on intervention/implementation strategy development [1, 2], we also present findings demonstrating relative value of each of three qualitative methods employed in the alpha test as they relate to overlap, divergence, and quality of information learned. Our discussion highlights the overall value of alpha testing as an intervention development approach in relation to our findings. We present our methods and findings separate from those of the subsequent beta testing phase to provide detailed guidance related to alpha testing that is currently missing from the intervention development literature. The methods described below provide a more robust description of the instruments (including the addition of an online questionnaire), protocols, and analysis approach employed in the alpha testing phase of the HFTAT than were provided in the previously published protocol article [11].

## Methods

### Participants

In order to collect the most informative data in a relatively short time window, we employed a purposeful sampling approach to select a small number of participants with a breadth of HF understanding and experience. We selected five providers from each of two large cities (*n* = 10), one with a high degree of successful HF implementation across its homeless service system and one without, to participate in the alpha test. We identified individuals with assistance from community partners in each of the cities. Regarding other pertinent characteristics of our sample, five participants were program directors and five were staff. Formal education was divided equally with five having a bachelor’s and five having a master’s degree. There was a range of experience providing housing services, with two participants having less than 1 year, two between 1 and 5 years, and the rest having more than 5 years of experience.

### Procedure and measures

We followed a pragmatic approach to data collection [26], utilizing methods that would provide the most exhaustive results with the fastest turnaround given the need to conduct this study phase in a relatively short period of time. While most alpha tests are carried out at the development site where participants are observed as they progress through the user experience, we used a remote, asynchronous approach, asking participants to complete the modules in a time and place of their choosing. Our data come from three sources.

Data were collected using multiple methods. We instructed participants to complete (1) a structured *user log* (see Additional file 1) as they worked through each module in real time. User logs are an approach often used to understand user experience of new technologies [27]. Our log was a paper-based instrument aimed at understanding user experience through the collection of the following information: technical issues experienced, questions and concerns regarding information or content, and any additional comments regarding the module participants might have.

After completing each module, participants were presented with (2) a *brief online questionnaire* (see Additional file 2) delivered using the Research Electronic Data Capture system [28]. The questionnaire comprised 12 Likert-type items (1 = “totally disagree,” 5 = “totally agree”) from the Training Satisfaction Rating Scale (some items were slightly reworded to better fit the context of the study), which has demonstrated construct validity and reliability established across 78 different training activities encompassing a wide variety of content areas [29], and 6 open-ended questions. Five of the open-ended questions corresponding to specific items asked participants “Why did you choose this rating?”, and a final open-ended question asked participants “Are there any other comments about the module you would like to make?”

We conducted (3) *focus groups* (approximately 2 h long) in each city within 2 weeks of all participants completing the modules. The goal of the focus groups was to assess the feasibility of the eLearning modules as a training component of the HFTAT. Areas of questioning included participant experiences with the training modules from the perspectives of the level and quality of content, training delivery, and aspects of content interaction. In addition, we queried the extent to which the modules aligned with their experiences of working in housing and asked participants how they imagined the modules might be used in implementation (see Additional file 3 for focus group guide). Two individuals in one city could not attend the focus group scheduled there, and we made accommodations for a single phone interview at a time convenient for both of them. Participants were compensated $100 for the time it took them to complete each module and $30 for focus group participation. The alpha testing process from initial consent to the final focus group took just over 5 weeks to complete. Copies of all instruments used in this study are included with the supplementary materials.

### Analyses

We first calculated descriptive statistics (medians and ranges) for each of the training satisfaction items to provide a basic understanding of participants’ satisfaction with the modules. We then transferred questionnaire and log data to word-processed documents and transcribed focus group audio files verbatim. Two analysts (EA and EQA) completed qualitative coding. Because we began with major areas of inquiry in mind to guide our analyses, we used a directed content analysis technique [30].

The first coding cycle began with independent initial readings of the data and identification of text comments relevant to the research questions, which formed the initial basis for the code list. Next, each analyst independently applied the code list, a combination of descriptive and simultaneous codes, to the data [31]. These types of codes use researcher-generated words to develop labels and allow for the same portion of data to be assigned multiple codes. In subsequent meetings, analysts compared their lists and data exemplars and returned to the data to apply them. Over several rounds, this led to a refined codebook with definitions for codes and sub-codes related to the type of feedback received from participants.

Using the codebook, each analyst individually pattern coded the entire dataset and together checked consistency in code application over several analysis rounds, discussing all discrepancies until consensus. The analysts then developed pattern codes into narrative description supported by data. Finally, the analysts used matrices to understand the quality and contribution of each data source to the resulting findings [31]. All qualitative analyses were supported by MAXQDA software [32].

## Results

We first present our overall findings as they relate to participants’ assessments of acceptability and feasibility, within which identification of bugs and technical concerns are highlighted. We then present our findings related to the unique contributions of each qualitative data source.

### Acceptability of the eLearning modules

Overall assessment of the modules by participants was highly positive in terms of both their relevance and quality of presentation. Table 1 presents medians and ranges for each question by module. Mean ratings (not shown) of the modules were high overall, ranging between 3.22 and 4.5. While individual ratings tended to stay above a neutral score, there were a few instances where participants demonstrated dissatisfaction.

The rest of our results will focus on findings from the qualitative data. Several participants expressed surprise that they enjoyed the modules, and one declared it the “best webinar I’ve sat through that dealt with harm reduction.” Participants viewed the quality of content quite positively, using words such as “spot-on,” “digestible,” and “great” to discuss it. In particular, they expressed appreciating the use of client narratives to exemplify covered content and found the stories to be realistic portrayals of clients they served. They referenced additional specific content areas they especially appreciated because they were either useful to their particular work or viewed as under-addressed in past trainings in which they had participated. Participants also appreciated the mixed-media delivery of content because the change from written to audio and back helped hold their attention. Despite this, there were comments from some that they would have preferred more audio content. Finally, almost unanimously, participants wanted more feedback on interactive activities, particularly in cases of quizzes where no feedback other than the correct answer to a question was provided and instances where they felt more explanation was needed when differences between multiple-choice answers were subtle. They also expressed wanting the ability to navigate back and forth to specific points in the module more easily, both so that they could stop and start the training and to review materials again.

**Table 1:** Median scores for answers to training satisfaction questions by module

Item	Module 1 (*n* = 9)	Module 2 (*n* = 10)	Module 3 (*n* = 10)	Module 4 (*n* = 10)
	Median (range)	Median (range)	Median (range)	Median (range)
1. In my opinion, the planned objectives of the module were met.	4 (4–5)	4 (3–5)	4 (4–5)	4 (4–5)
2. The issues were within as much depth as the length of the module allowed.	4 (4–5)	4 (2–5)^a^	4 (3–5)	4 (2–5)^a^
3. The length of the module was adequate for the objectives and content.	4 (3–5)	4 (2–5)^a^	4 (2–5)^a^	4 (2–5)^a^
4. The method was well-suited to the objectives and content.	4 (4)	4 (4–5)	4 (3–5)	4 (3–5)
5. The method used enabled me to take an active part in training.	4 (4–5)	4 (4–5)	4 (2–5)^a^	4 (3–5)
6. The training enabled me to share professional experiences with colleagues.	3 (2–5)^b^	4 (2–5)^b^	4 (3–5)	4 (3–5)
7. The information in the modules was realistic and practical.	4 (4)	4 (4–5)	4 (4–5)	4 (4–5)
8. The documents linked to the module were of good quality.	4 (3–4)	4 (3–5)	4 (1–5)^c^	4 (3–5)
9. The training context was well-suited to the training process.	4 (3–4)	4 (3–5)	4 (4–5)	4 (3–5)
10. The training received in this module is useful for my specific job.	4 (3–5)	4 (3–5)	4 (3–5)	4 (3–5)
11. The training in this module is good for my personal development.	4 (3–5)	4.5 (3–5)	4 (4–5)	4.5 (3–5)
12. The training in this module merits a good overall rating.	4 (4–5)	4 (2–5)^a^	4 (4–5)	4 (4–5)

Items scored on scale from 1 = “totally disagree,” 5 = “totally agree”

^a^Item received one score of 2 (i.e., disagree)

^b^Item received two scores of 2 (i.e., disagree)

^c^Item received one score of 1 (i.e., totally disagree)

Regarding the modules’ appearance, participants were largely ambivalent regarding the use of static images to represent individuals who were speaking. However, they recognized video recordings of non-actors can feel awkward and stilted. Additionally, some participants discussed a perceived ethnic mismatch between individuals portrayed in the modules and their own client bases. They also found technical directions to be lacking: these included a lack of consistency and clarity regarding module navigation and need for clearer indication when the module directs learners to a website outside of the “closed” eLearning environment (e.g., the CoP). They viewed the quality of additional resources as equally positive, referencing the possibility of returning to them at a later date.

The CoP, which is hosted on a website separate from the modules and hyperlinked to specific points in the training, was under-utilized. While participants were positive regarding the idea of the forum hosted in the CoP because it could provide individuals with exposure to other viewpoints and expand their professional networks, they felt reticent to use it because of uncertainty regarding “who was out there” (i.e., who could read their comments) and “whether this venue was safe.” Since they were completing the training modules in addition to performing their jobs, participants also described skipping CoP activities, with the thought they would return later when they had more time. Some participants indicated they did not use the CoP because they were simply confused by what they were supposed to do. Despite these issues, participants felt the CoP was a potentially positive feature that could facilitate professional development with greater clarification and ease of use. In particular, participants felt it might be useful in the longer term when formal implementation assistance had ended as a way to seek advice, resources, and troubleshoot. They additionally felt the CoP needed to be moderated somehow in order to ensure the environment remained civil.

Similar confusion existed regarding the digital badges. Some participants who did not use online professional or social networking sites did not understand what they were for, where they went, or what aspects of their performance on the module would be displayed with the badges. Since they were “not a real credential” recognized by an official body, a number of participants thought the badges might be received better if offered as positive reinforcement at shorter intervals.

### Feasibility of the eLearning modules as a training strategy

Participants saw the training as valuable for *first exposure to ideas* about HF. They identified a number of different potential audiences, including staff learning about HF in onboarding or transitioning from a treatment-based approach, other organizations not working in HF but which nonetheless might need to understand the work of a HF organization, and potential funders of HF programs. Participants also identified learning related to HF facts and practices and the training’s potential to challenge preconceptions and ideas as valuable. While suggested strategies for addressing issues that arise in HF programs were appreciated, most participants expressed eLearning modules presented inherent limitations to developing the “muscle memory” required to use skills. Nonetheless, they also clearly recognized where they were in their own practice and imagined how they might apply information learned in relation to supervisors, subordinates, landlords, or residents. They saw this vision as the first step to building new skills. One example of this related to being more persuasive in discussing HF with skeptics: participants suggested that the modules presented a “flavor” and vocabulary relating to HF that helped them envision a conversational tone they thought might broaden peoples’ visions of HF.

One significant feasibility issue was hosting of the CoP on a separate website. This was annoying and problematic because participants had to log in by creating a separate account or using their Facebook or Google account. A second and more substantial problem was that some employer firewalls prevented them from logging onto the CoP on their work computers.

In addition to using the eLearning modules individually, participants believed they could be employed as a group to build discussion around challenges and strategies. People with leadership and supervisory roles indicated the modules would be a good alternative to conducting HF trainings themselves—something they did not feel they had sufficient expertise to do—and use of the modules would allow them more freedom to play a role more akin to a peer with their subordinates. Supervisors imagined several ways the modules might be of use in that function: using the training modules as starting points for discussion, helping staff avoid learning “the hard way,” and providing refreshers regarding specific content. Since most of the conversation about supervision was framed as supporting staff, they appreciated materials related to facilitating supervision and communication. Finally, they believed free or inexpensive modules would present a valuable alternative to “expensive experts” who might otherwise have to be contracted to support HF implementation, which they suggested many housing agencies could not afford.

While participants stated the modules would serve a useful role in implementation efforts, they suggested additional strategies and materials to further improve feasibility. These focused especially on what they believed administrators would need to hear to be convinced, including a desire for additional information on liability, costs, and benefits as compared to treatment-based programs, and the specific challenges related to each type of housing setup (i.e., multiple site or single site housing projects). They also wished for materials aimed at housing residents, such as materials to help residents understand what HF is and is not.

Finally, participants discussed the modules in terms of their ability to promote general workplace effectiveness and professional empathy. While certain modules were aimed at specific roles within a housing program, all participants felt learners should complete all modules, regardless of their roles, because it facilitated understanding of the work and challenges each person would have. They believed this professional understanding was vital to making a HF program work.

### Contribution of each data source

Table 2 shows the relative presence of themes and codes in each qualitative data source. Each data source had strengths and weaknesses for our alpha testing phase. For instance, references to specific “bugs” at particular points of any given eLearning module (typo/grammar problem, visual layout quality, audio quality, mobile interface) came almost exclusively from the logs or the questionnaires tied to each module. While concerns relating to the quality of the audio in the modules, noted inconsistencies in presentation, ways participants interacted with the material, and navigation issues were present across sources, they were most salient in the logs. The open-ended questionnaire items associated with each module provided mostly user satisfaction data, with some data related to momentary inspirations about how the module’s information might be applied in daily work. Focus groups provided the highest quality data regarding how the intervention could best be used as an implementation strategy. Participants identified challenges to quality or usability which were more complex than noting bugs, and items specifically related to *this training* rather than those related to eLearning in general. Focus groups also helped us understand how intended participants would employ the implementation strategy in ways we had not always predicted. For instance, we had not envisioned the kind of group use of the modules to promote ongoing discussion of HF in supervision activities described above. The data sources were also useful as a group, as comments provided in logs were often brief and hard to interpret; without the focus groups, these would have been less useful. Interestingly, we learned many participants used the logs to prepare for focus group discussions, reviewing their notes to remind themselves of things they wanted to bring up in more detail.

**Table 2:** Codes and themes from each data source

Theme	Journal/log	Open-ended questionnaire response	Focus group
Content			
General compliments	✓	✓	✓
Self-reflection	✓−	✓	✓
Quality of content	✓	✓	✓
Level of information	✓	✓	✓−
Range of information	✓−	✓	✓+
Quality of resources	✓	✓	✓−
Congruence with experience and prior knowledge	✓	✓	✓
Congruence with expectations	✓	✓	✓
New ideas	✓	✓	✓
Delivery			
Active learning	✓−	✓	✓
Balance of audio/written	✓	✓	✓
Visual appearance/display	✓	✓	✓+
Clarity of direction: content	✓+	✓	✓
Clarity of direction: feedback on interactive responses	✓	✓	✓
Clarity of direction: technical instructions	✓	✓	✓+
Desire for back-navigation	✓	✓	✓
Mobile interface	✓		✓
Point of view		✓	✓+
Uses			
First exposure to ideas	✓−	✓	✓
Learn content	✓−	✓	✓
Learn strategies/skills	✓	✓	✓
Implement HF	✓−	✓	✓+
Training	✓−	✓	✓
Leadership/supervision		✓	
General workplace effectiveness		✓	✓−
Persuasion/general conversation	✓	✓	✓
Professional empathy			✓
Technical issues			
Access to external resources	✓		
Audio quality	✓+	✓	
Visual layout quality	✓		✓
Inconsistencies	✓+	✓	✓
Interaction and navigation/pacing	✓+	✓	✓
Typo/grammar problem	✓		
Badges/credentialing			✓
Community of practice	✓	✓	✓+
Other	✓−	✓	✓+

+ Means especially present in this source; − means minimally present in this source

## Discussion

As expected, alpha testing provided valuable information regarding acceptability and feasibility of the HFTAT eLearning modules. Participants found the training to be enjoyable overall. Most importantly, the data support our original assumptions regarding the value of using case-based narratives in training activities, reinforcing the potential value of narrative storytelling as an approach to facilitate learning [18–21], what has been referred to as dynamic training in the implementation literature [3, 33]. Findings also demonstrate that the mixed-media (e.g., audio, video, images, and text) approach used to deliver information, the activities, and breaking up of content over multiple lessons kept participants attentive and engaged, which supports current theories regarding effective learning [22, 34]. Participant recognition of eLearning as a potentially more affordable approach to training also hints at its potential attractiveness as an implementation strategy, as cost-effectiveness is an important factor when considering intervention scalability [35]. A low-cost, distance-based implementation strategy is likely to be even more attractive to organizations located outside major metropolitan areas that may not have resources to bring trainers to them. Anecdotally, the principal investigator has already been contacted by one rural organization seeking affordable Housing First training opportunities after they learned about our study.

While overall opinions were positive, attention must also be paid to problematic issues noted in order to improve training effectiveness moving forward. Most importantly, the data support prior evidence of training as necessary, but insufficient implementation strategy and the need to understand how multiple discrete strategies may or may not complement one another [1, 36–39]. One example is participants’ recognition that the training was not suitable for practicing and fully developing skills necessary for HF practice. Champagne et al. [39] had similar findings in their assessment of an online training strategy. They conclude that a combination of socialization and communication between multiple actors are necessary for converting improved knowledge and attitudinal changes affected by training to routinized skills necessary for optimal intervention delivery. Indeed, we developed the HFTAT as a multifaceted strategy specifically to address training limitations such as these: the goal of the CoP is to encourage interaction among practitioners, and the technical assistance to be added in the pilot stage will provide a space for administrators and champions to discuss issues arising during implementation with expert trainers and as a group.

Despite the above stated goal of the CoP, it seems as though it and the digital badges may not be as effective as we believed they would be when conceptualizing the study. Normalization process theory [22], which aims to explain the processes through which complex interventions become routinized, provides some indication for why these components might have failed to be as effective as expected. First, participants’ lack of understanding regarding the CoP’s purpose and privacy concerns, related to the open online environment, demonstrate a lack of interactional workability (i.e., the interplay between people or people and systems they use to carry out the intervention) between the learner and the CoP, a problem that has been noted in previous research [40]. Second, contextual integration (i.e., relation between the organizational setting and the intervention) is lacking for both the CoP and digital badges. For instance, online security protocols and time constraints within the organization acted as a significant barrier to CoP use for some, while the badges were not recognized as a legitimate form of credentialing by the organization or participants’ professions. This drawback of digital badges has been discussed in previous literature [41, 42], and it has been noted that badges will likely not be widely adopted until large authoritative organizations legitimize them [43]. Despite identified problems, we decided to keep the CoP and digital badges for the subsequent pilot. However, we did make modifications to encourage greater engagement with these aspects including privatizing the CoP forums so they can only be accessed by trainees and providing more detailed description of the badges. An additional incompatibility between the context and the intervention noted by participants was the mismatch between the images of people represented in the training and the people they served. Foreseeing this as a possible issue due to the lack of diversity we encountered in commercial stock photos, we did develop a number of our own images to represent a wider range of races, ethnicities, and ages. We will likely need to develop more to include in future versions of the modules when time and finances allow.

Alpha testing also helped us identify and fix a number of problems with the modules that would have been more difficult to deal with had they not been noticed until the subsequent pilot. We made the following modifications based on our findings: fixing of technical issues and bugs (e.g., problematic slide progression, hyperlink issues, and spelling and grammar problems), more feedback and explanation at the end of activities, inclusion of summary lessons within the modules that participants can download upon completion, and including hyperlinks to another version of the modules users can use to review. Technical errors such as these could have negatively impacted pilot results through their effect on the participant experience. Maintenance required to correct errors such as these might also negatively impact participants’ experiences if they are unable to access modules when maintenance is being performed.

Finally, our findings demonstrate the strength of using multiple methods for the early stages of intervention design. While we did collect some quantitative data through the satisfaction surveys, the most useful data were qualitative in nature. This supports the limited literature on intervention development methods, which generally stresses the usefulness of qualitative approaches for assessing acceptability and feasibility and for understanding the specific circumstances in which the intervention will be used for purposes of improvement [1, 44, 45]. In relation to our study, logs and questionnaires reflected participants’ immediate reactions to the modules and were most useful for identifying specific technical issues, while focus groups allowed participants to expand thinking, describe how module content integrated with their professional experience, and explore their potential usefulness of eLearning as an implementation strategy. Focus group data also confirmed and expanded on issues identified through the other methods, a specific example being reasons behind participants’ lack of interaction with the CoP. Finally, our methodological findings also demonstrate the usefulness of remote, asynchronous methods for collecting usability data. While they may not be suitable for all studies, remote, asynchronous methods can save time and expense when compared to the traditional approach of observing individuals as they use the technology of interest [46].

Despite the very rich information to be obtained through multiple methods, researchers must consider burdens related to each method when designing protocols. For instance, each of our methods required different demands of participant and researcher time. For participants, logs were undoubtedly the most time intensive and mentally demanding, as they had to move back and forth to them as they worked through each module. Researchers experienced different burdens related to instrument development, data collection, and analysis. While the logs were straightforward in development and analysis, questionnaire development was more time consuming than analysis of their resulting data. Focus groups required the greatest time and intellectual investment in terms of analysis because of transcription of audio recordings and the resulting volume of raw data. As such, researchers should carefully consider potential burdens in combination with the particular benefits of specific data sources in relation to what they want to learn [47]. In hindsight, we could have reduced data collection burden on participants by not using the questionnaire, as the information received was largely redundant with the other two sources.

Regarding limitations, our findings are far from statistically generalizable given our qualitatively driven approach; however, this is not the purpose of an alpha test. What we did achieve was a relatively quick and effective testing of the modules that allowed us to move quickly to the next study phase. Additionally, the common themes identified across sources (see Table 2) demonstrate trustworthiness and strengthen evidence contributing to validity [26]. While remote, asynchronous approaches to data collection likely saved us time and resources, large variations in the amount of detail provided in logs suggests employing an observational approach. This approach, more typical of usability studies, might have yielded equally rich data across participants that could have assisted us in identifying more issues.

## Conclusions

Alpha testing was a valuable step in the development of the eLearning modules that provided us with preliminary validation of our overall approach and an understanding of issues that would have been more difficult to address at a later stage of the study. Each method of data collection employed provided some level of unique information that contributed to a more holistic understanding of the intervention when combined. As a result, we were able to improve the modules prior to pilot testing of the entire HFTAT. Alpha testing of this sort may not be a necessary or appropriate stage for all interventions, and the decision whether or not to employ it is up to the developers. That said, our results demonstrate it is a useful process to go through for any intervention that relies on technology that can suffer from technical issues or bugs. Researchers wishing to alpha test interventions prior to piloting should consider the unique benefits of different approaches to data collection and balance this with the need to minimize burdens for themselves and their participants.

In terms of next steps, pilot testing of the entire HFTAT began in January of 2016 and will continue into the summer of 2017. The focus of the pilot is to understand the utility of the strategy for affecting individual staff attitudes and knowledge and the organization’s fidelity to HF practice. Our eventual goal after the pilot is to conduct a larger study to test the efficacy of separate components of the HFTAT (e.g., eLearning alone, technical assistance alone, eLearning combined with technical assistance) in order to understand their relative value. Both of these studies will provide insight into the value of multifaceted versus discrete implementation strategies and the value of eLearning interventions, two areas in need of investigation [3, 10].

## Additional files

Additional file 1: Alpa test user log. (DOCX 17 kb)

Additional file 2: Alpha test online module survey. (DOCX 18 kb)

Additional file 3: Alpha test focus group guide (DOCX 18 kb)

## Abbreviations

CoP: Community of practice
HF: Housing First
HFTAT: Housing First Technical Assistance and Training

## Footnotes

1 We developed the modules using Articulate Storyline eLearning software and delivered them over the same company’s learning management system (Articulate Global Inc., New York, NY; https://articulate.com/).

2 Much of the work on digital badges has been advanced by the Macarthur Foundation, and more information on digital badges can be found on their website (https://www.macfound.org/programs/digital-badges/).
