# Supplementary material for: Alpha test results for a Housing First eLearning strategy: the value of multiple qualitative methods for intervention design
Source: Pilot Feasibility Stud. 2017 Oct 31;3:46. doi: 10.1186/s40814-017-0187-y (PMC5663117; doi:10.1186/s40814-017-0187-y)
Supplement: Supplementary file 1 — Alpa test user log. (DOCX 17 kb) [file 40814_2017_187_MOESM1_ESM.docx]

**User Log**

Use this form to record your experience, impressions, concerns, and suggestions regarding each HFTAT lesson as you work through it. Please keep the document next to you as you interact with the training content (rather than filling it out after the lesson is completed).

|  | Comments/Ratings |
| --- | --- |
| Technical issues experienced (please provide slide number, title, or time in presentation problem occurred if possible.) |  |
| Questions or concerns regarding information or content |  |
| How long did it take you to work through the lesson? |  |
| Any other comments about the module you would like to make? (feel free to write on the back of this page or attach another sheet of paper if needed): | |
