# Supplementary material for: Alpha test results for a Housing First eLearning strategy: the value of multiple qualitative methods for intervention design
Source: Pilot Feasibility Stud. 2017 Oct 31;3:46. doi: 10.1186/s40814-017-0187-y (PMC5663117; doi:10.1186/s40814-017-0187-y)
Supplement: Supplementary file 2 — Alpha test online module survey. (DOCX 18 kb) [file 40814_2017_187_MOESM2_ESM.docx]

**Training Satisfaction Questionnaire^[[1]](#endnote-1)^**

| Please answer questions 1-12 to the right using the following scale:  1 = totally disagree  2 = disagree  3 = neutral  4 = agree  5 = totally agree | 1. In my opinion, the planned objectives of the module were met. _____ 2. The issues were within as much depth as the length of the module allowed. _____ 3. The length of the module was adequate for the objectives and content. _____ 4. The method was well suited to the objectives and content. 5. The method used enabled me to take an active part in the training. _____    - Why did you choose this rating? 6. The training enabled me to share professional experiences with colleagues. _____ 7. The information in the module was realistic and practical. _____  - Why did you choose this rating?  1. The documents linked to the module were of good quality. _____ 2. The training context was well-suited to the training process. _____ 3. The training received in this module is useful for my specific job. _____    - Why did you choose this rating? 4. The training in this module is good for my personal development. _____    - Why did you choose this rating? 5. The training in this module merits a good overall rating. _____    - Why did you choose this rating? |
| --- | --- |
| Are there any other comments about the module you would like to make? | |

1. Questions adapted from Holgado Tello FP, Chacón Moscoso S, Barbero García I, Sanduvete Chaves S. Training satisfaction rating scale: development of a measurement model using polychoric correlations. Eur. J. Psychol. Assess. 2006;22:268–79. [↑](#endnote-ref-1)
