# Supplementary material for: Alpha test results for a Housing First eLearning strategy: the value of multiple qualitative methods for intervention design
Source: Pilot Feasibility Stud. 2017 Oct 31;3:46. doi: 10.1186/s40814-017-0187-y (PMC5663117; doi:10.1186/s40814-017-0187-y)
Supplement: Supplementary file 3 — Alpha test focus group guide (DOCX 18 kb) [file 40814_2017_187_MOESM3_ESM.docx]

Thank you for agreeing to participate in tonight’s discussion of the HFTAT program. Let’s begin by introducing ourselves. We’ll use first name and occupation – specifically, I’d like to know whether your job is mostly program staff, administrative, or housing systems level. When this audio is transcribed, we will remove your names. I’ll start. I’m ____, and I am a researcher.

…

Great, thank you everyone. This evening we’d like to discuss the training modules for Housing First implementation you’ve been working with. We’ll spend a little time talking about the content in them, the ways they are presented, and their ability to help organizations implement Housing First practices. Then we’ll move on to other aspects of the training modules you may want to discuss. We really appreciate your willingness to discuss your experiences, because your feedback will help us to understand the perspective of users of the training modules.

------------------------------------------------------------------------------------------------------------------------------------------

1. *Let’s begin by talking a little about the content of the modules themselves.* Could you tell me about how you viewed the level of the information presented?

Probe: How do you think that might affect user perception of the training modules?

1. A focus of the modules is to discuss common barriers to implementing Housing First practices. What is your opinion about the range of barriers presented?

Probe: Can you think of implementation challenges which are not addressed?

1. Do you have thoughts or opinions about specific modules?

*Thanks for your input on the content of the trainings. I’d like to move now to discussing the method/interface/style in which the training modules are delivered.*

1. *We know that just presenting people with facts isn’t enough to change values-based opinions. This is why stories and scenarios were used in the training modules to talk about Housing First, harm reduction, and barriers to implementing Housing First practices.* How did you feel about the stories and scenarios which were used in the training modules?

Probe: Style, variety used.

Probe: How do you think these are likely to be perceived by program staff and administrators?

1. *As part of the modules, users complete short activities, like quizzes, making choices based on scenarios presented, or answering a question about their own experiences.* What did you think of those activities?

Probe: Style, variety used.

Probe: How do you think these are likely to be perceived by program staff and administrators?

1. *A final aspect of the way the training modules are delivered which I’d like to discuss is the online community of practice.* What was your experience with that aspect of the training modules?
2. *Sticking with the online community of practice for a moment,* what do you think about what it might be used for by people completing the training modules?

Probe: sharing ideas, understanding local context, situations, needs, possible solutions, connections to national resources

1. *One more question about the online community of practice.* What is your opinion about the use of online communities of practice outside of a training setting?

*The information you are providing is really valuable in thinking about these training modules. Another thing we’re interested in learning about is possible outcomes of training for users of the training.*

1. What do you think about the ability of the training modules to help users learn new skills and strategies?

Probe: Differences by occupation (program staff, admin, systems person)

1. *Users of the training modules earn digital badges which certify their completion of the training module.* What is your opinion about digital badges?

Probe: badges as motivators to trainees; usefulness as a credential

1. *I just have one more area I’d like to hear about from you.* What is your opinion of the ability of the training to help with implementation of Housing First practices?

Probe: feasibility, effectiveness

1. *This training program consists of the modules we’ve been discussing as well as technical assistance.* What is your view about whether the modules could stand alone without the technical assistance?
2. Is there anything else you’d like me to know about your experience with the training modules?

*Thank you for talking to me about the HFTAT program. The insights you’ve shared will be helpful to improving the training for others hoping to implement Housing First practices.*
